# Case report of pharmacokinetic analysis of continuous intravenous infusion of fentanyl in a patient with severe burn: burn shock stage complicates pain management

**DOI:** 10.1186/s40780-024-00363-9

**Published:** 2024-07-16

**Authors:** Takafumi Nakano, Yasuhisa Oida, Shinichi Morimoto, Kentaro Muranishi, Soichiro Ushio, Takuya Yamashina, Masanobu Uchiyama, Kenichi Mishima, Kiyoyuki Kitaichi, Yoshihiko Nakamura, Koichi Matsuo

**Affiliations:** 1 Department of Oncology and Infectious Disease Pharmacy, Faculty of Pharmaceutical Sciences, Fukuoka University, 8-19-1 Nanakuma, Jonan-ku, Fukuoka, 814-0180 Japan; 2 Laboratory of Pharmaceutics, Department of Biomedical Pharmaceutics, Gifu Pharmaceutical University, 1-25-4 Daigaku-nishi, Gifu, 501-1196 Japan; 3 Department of Emergency and Critical Care Medicine, Fukuoka University Hospital, 7-45-1 Nanakuma, Jonan-ku, Fukuoka, 814-0180 Japan; 4 Department of Physiology and Pharmacology, Faculty of Pharmaceutical Sciences, Fukuoka University, 8-19-1 Nanakuma, Jonan-ku, Fukuoka, 814-0180 Japan

**Keywords:** Fentanyl, Burn injury, Continuous intravenous infusion, Burn shock stage

## Abstract

**Background:**

Fentanyl is widely used as an analgesic and sedative for patients with severe burn injuries in intensive care units. However, pharmacokinetic (PK) data for fentanyl, particularly for continuous intravenous infusion during the acute phase of burn injuries, are limited. Here, we report the clinical course and changes in blood fentanyl concentrations during the acute phase in a patient with severe burns treated with continuous intravenous infusion of fentanyl.

**Case presentation:**

A woman in her 40s, with burns caused by a gas cylinder explosion, was transported to our hospital. The patient had burn wounds on face, neck, shoulders, and all four extremities, with a total burn area of 39.0%. For pain relief, the patient received a continuous infusion of 0.01 mg/mL fentanyl (20–30 µg/h) with a target blood concentration of 1.0–1.5 ng/mL, but continued to suffer from pain due to burning during the acute phase. We measured the blood fentanyl concentrations and found that all concentrations obtained during the acute phase were subtherapeutic. Notably, during the burn shock stage, blood concentrations of fentanyl were 0.50 ng/mL on day 1 and 0.66 ng/mL on day 2, indicating that the blood concentration did not rise sufficiently for the dosage. From days 0 to 2, the patient was administered a massive fluid load for burn shock. After the burn shock stage resolved, fentanyl concentrations gradually approached the target range, and the pain rating scale improved, even though the fentanyl administration rate remained unchanged (30 µg/h).

**Conclusions:**

Major changes in the fluid volumes of body compartments that occur with large burns might increase the volume of fentanyl distribution, thereby lowering its concentration when a standard dose is administered. Our findings indicate that the PK of fentanyl in patients with severe burns can be substantially affected, especially during the shock phase, implying the importance of titrating analgesics for clinical efficacy in the acute phase.

**Supplementary Information:**

The online version contains supplementary material available at 10.1186/s40780-024-00363-9.

## Introduction

Burn injuries are one of the most painful traumas, have long-term physical and psychological impacts, and are extremely difficult to manage. Severe burns require intensive management of both long-term pain due to burns and short-term acute pain due to procedures such as dressing changes [1]. Opioids have been established as a basic pain management therapy in patients with severe burns because of their excellent efficacy in providing adequate analgesia [2–4]. Fentanyl, a synthetic opioid, has both efficacy and potency for pain and is known to have a lower risk of side effects than morphine [1, 2]. Therefore, fentanyl is widely used as an analgesic and sedative in patients with severe burn injuries in intensive care units (ICU).

Initial burn shock is a hypodynamic and hypovolemic state, with rapid fluid loss from the intravascular space and decreased cardiac output, which lasts for the first 24–48 h [5, 6]. Therefore, organs in the burn shock stage require large volumes of fluid infusion, which rapidly dilutes plasma proteins and expands the intravascular volume. Moreover, after successful fluid resuscitation after burn shock, a hypermetabolic state occurs with increased cardiac output and reduced systemic vascular resistance [7, 8]. Increased cardiac output during a hypermetabolic state leads to increased hepatic, splanchnic, and renal blood flow, resulting in increased metabolic and renal clearance of drugs [7, 8]. Therefore, patients with severe burns have the potential to influence the pharmacokinetics (PK) of many drugs, including fentanyl, owing to pathophysiological changes and therapeutic interventions (e.g., hemodynamic alterations, organ failure, and capillary leak).

An intravenous bolus of fentanyl in patients with burns has been reported to result in lower blood concentrations than in patients without burns [5, 9, 10]. In contrast, continuous intravenous infusion of fentanyl has recently been recommended for burn pain management [4], but there are few reports on PK alterations in patients with burns treated with continuous intravenous fentanyl infusion. Here, we report the clinical course and changes in blood fentanyl concentrations during the acute phase in a patient with severe burns treated with continuous intravenous infusion of fentanyl.

### Case

The patient was a woman in her 40s with a history of hypertension, hyperthyroidism, and insomnia. The patient was transported to our hospital with burns caused by a gas cylinder explosion. On admission, the level of consciousness was I-1 on the Japan Coma Scale and 15 on the Glasgow Coma Scale. Her vital signs were as follows: blood pressure, 121/84 mm Hg; respiratory rate, 20 breaths/min; pulse rate, 106 beats/min; and SpO_2_, 99% (O_2_ was 10 L). The admission weight of patient was 45 kg, and burns had spread to the face, neck, shoulders, and all four extremities, with a total burn area of 39.0% (14% third-degree and 18% second-degree; burn index, 23; prognostic burn index, 76). Based on her consciousness, respiratory status, and bronchoscopic findings, intubation was not performed. Table 1 shows the laboratory values at admission. Organ failure, such as liver or renal dysfunction, was not observed. The patient underwent fluid resuscitation based on the method of Baxter (7644 mL) during the first 24 h, and then the fluid volume was adjusted according to the urine volume. Although accurate assessment of blood pressure was difficult during the burn shock stage owing to pain-related body movements, the patient’s systolic blood pressure (sBP) was maintained in the range of 90–120 mmHg during the burn shock stage on days 1 and 2. From day 3, after the burn shock stage had resolved, the sBP was maintained in the range of 110–140 mmHg. Therefore, during the treatment period, her hemodynamic parameters, including blood pressure and pulse rate, were maintained.

**Table 1:** Results of the blood test performed on hospital arrival

[Blood count / biological]				[Blood gas/coagulation and fibrinolytic system]		
WBC	8.3	× 10^3^/µL		pH	7.353	
RBC	393.0	× 10^4^/µL		PCO_2_	42.3	mmHg
Hemoglobin	12.5	g/dL		PO_2_	285.0	mmHg
Platelet	23.3	× 10^4^/µL		HCO_3_^-^	22.9	mmol/L
Albumin	4.5	g/dL		BE	−2.0	mmol/L
CRP	0.01	mg/dL				
BUN	22.0	mg/dL		PT-INR	1.0	
Cr	1.2	mg/dL		APTT	22.5	S
Na	142.0	mmol/L		FDP	3.0	µg/mL
K	3.1	mmol/L		D-dimer	< 0.5	µg/mL
Cl	104.0	mmol/L				
Total bilirubin	0.5	mg/dL				
AST	40.0	IU/L				
ALT	30.0	IU/L		[Score]		
ChE	347.0	IU/L		APACHE-II	7	Points
LDH	267.0	IU/L		SOFA	2	Points
CK	572.0	IU/L		ISS	16	Points
Glucose	104.0	mg/dL				
Lactic acid	33.0	mg/dL				

ALT, alanine aminotransferase; APACHE, acute physiology and chronic health evaluation; APTT, activated partial thromboplastin time; AST, aspartate aminotransferase; BE, base excess; BUN, blood urea nitrogen; ChE, cholinesterase; Cr, creatinine; CK, creatine kinase; CRP, C-reactive protein; FDP, fibrin degradation product; ISS, injury severity score; LDH, lactate dehydrogenase; PT-INR, prothrombin time-international normalized ratio; RBC, red blood cell; SOFA, sequential organ failure assessment; WBC, white blood cell

**Fig. 1:**
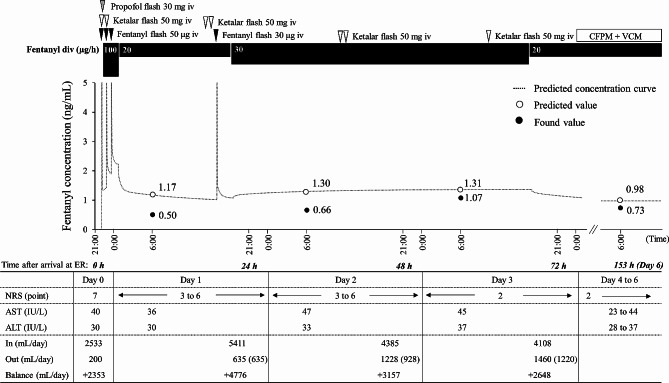
Clinical course of the patient The black points indicate the observed values, and the white points indicate the predicted values. The predicted concentration curves were analyzed using fentanyl dose simulation based on Shefer’s pharmacokinetic parameters. The number in parentheses in the Out item indicates the urine volume (mL/day) ALT, alanine aminotransferase; AST, aspartate aminotransferase; CFPM, cefepime; div, drip infusion into vein; ER, emergency room; iv, intravenous injection; NRS, numerical rating scale; VCM, vancomycin

The numerical rating scale (NRS) score for pain assessment was 7 points immediately after admission. The patient’s clinical course and pain management since admission are shown in Fig. 1. For pain relief, the patient received a continuous infusion of 0.01 mg/mL fentanyl (100 µg/h) after the fentanyl flush (50 µg). Three hours after starting fentanyl infusion, the rate of continuous infusion of fentanyl was adjusted to 20 µg/h. The rate of infusion was determined by fentanyl dose simulation based on Shefer’s PK parameters [11] to achieve a target blood concentration of 1.0–1.5 ng/mL. On day 1, the NRS score remained between 3 and 6 points, and pain control was inconsistent, even though fentanyl blood levels had reached a steady state. Thus, before day 2, fentanyl administration rate was temporarily increased from 20 µg/h to 30 µg/h. However, the NRS score remained between 3 and 6 points on day 2, and pain management was difficult. After this, the fentanyl administration rate was kept constant at 30 µg/h to avoid the risk of respiratory depression, as the patient was not intubated during the treatment period. The main therapeutic interventions on days 1 and 2 were massive fluid loading, analgesia with fentanyl, administration of a proton pump inhibitor, and debridement; no deep sedation was used during the treatment period.

On day 3, when the burn shock stage resolved, the NRS was 2 points, and pain control improved. Moreover, fentanyl administration rate was able to be reduced from 30 µg/h to 20 µg/h. From that day onward, the NRS remained 2 points and complaints of pain decreased, without changing the fentanyl administration rate. The main therapeutic interventions from days 3 to 7 were analgesia with fentanyl, administration of proton pump inhibitors and antibiotics, and debridement. Moreover, during the NRS assessment period, the only analgesics used were fentanyl and ketalar, as shown in Fig. 1.

To investigate the cause of difficulty in controlling pain during the acute phase, serum fentanyl concentrations were measured on days 1, 2, 3, and 6 using liquid chromatography-tandem mass spectrometry (LCMS-8045 triple quadrupole mass spectrometer; Shimadzu, Japan). In our measurement methods, no background peaks interfering with the fentanyl peak were observed. Moreover, fentanyl showed good linearity with an R^2^ value > 0.99 in the range of 0.05–1 ng/ml serum concentration, and the results were reproducible (supplementary Fig. 1). The patient’s serum fentanyl concentrations were as follows: 0.50 ng/mL on day 1, 0.66 ng/mL on day 2, 1.07 ng/mL on day 3, and 0.73 ng/mL on day 6. We found that the fentanyl concentrations on days 1 and 2, when the pain was not controlled, were only approximately 50% of the predicted concentrations. However, after the burn shock phase resolved (after day 3), serum fentanyl concentrations gradually approached the target range, even though the fentanyl administration rate from day 2 to day 3 remained unchanged. And the NRS score improved from day 3.

Written consent for the publication of this case report was obtained from the patient as per the Guidelines for Privacy Protection in Medical Papers and Conference Presentations, including Case Reports (Japan Surgical Society) [12]. This case report was not subjected to formal review by the Ethical Review Board of Fukuoka University Hospital.

## Discussion and conclusions

Factors influencing the PK of fentanyl include concomitant drugs that inhibit or induce drug metabolic pathways, impaired organ function, body weight, and age [9, 13, 14]. Patients with burns develop pathophysiological conditions that affect drug absorption, distribution, metabolism, and elimination [10]. For example, the volume of distribution (Vd) can be changed by fluid resuscitation, severe blood loss, transfusions, or extreme alterations in body weight. These phenomena are commonly observed during the critical care of patients with severe burns. Moreover, patients with severe burns receive more than 20 different drugs during hospitalization, many of which may affect the PK levels of fentanyl [15]. Therefore, PK data for fentanyl during the acute phase of burn injuries are limited. This is because it is difficult to clarify fentanyl PK in patients with severe burns owing to many factors, such as pathophysiological changes and therapeutic interventions.

This report describes the clinical course of a patient with severe burns during the acute phase and the changes in blood concentrations when fentanyl was continuously infused intravenously. In this patient, the rate of continuous intravenous infusion of fentanyl was nearly constant, except for the initial loading dose. Additionally, concomitant drugs that could affect fentanyl metabolism, such as cytochrome P450 (CYP) 3A4 inhibitor [16], were not used. Therefore, this case was considered suitable for clarifying the relationship between changes in blood fentanyl concentrations and pathophysiological changes during the acute phase of burn injury. Our observations demonstrated that blood fentanyl concentrations decreased during the burn shock stage and gradually increased to the predicted values after fluid resuscitation. Kaneda et al. and Han et al. previously revealed PK data after bolus administration of fentanyl in adult patients with severe burn injury [5, 10]. However, the pain of severe burns is not temporary but continues for a long time, which is a serious issue for patients. Therefore, in clinical practice, the severe burn pain is often managed through continuous intravenous infusion of fentanyl rather than bolus administration alone. This is the first report to show the changes in blood fentanyl concentrations through continuous intravenous infusion and clinical course in a single case.

The blood concentration required for fentanyl to exert an analgesic effect depends on the disease state and severity, but a blood concentration range of 1–2 ng/mL is recommended [9, 17–19]. In contrast, fentanyl concentrations > 2.0 ng/mL increase the incidence of respiratory depression in patients not receiving ventilator management [18, 19]. Therefore, the patient received a continuous infusion of fentanyl with a target blood concentration of 1.0–1.5 ng/mL based on the results of the fentanyl dose simulation to avoid respiratory depression. However, we measured the blood concentrations of fentanyl and found that the blood concentrations of fentanyl were only approximately 50% of the predicted values on days 1 and 2, indicating that the blood concentration did not rise sufficiently with the dosage. Several physiological factors, such as increased Vd, hypermetabolism, and CYP3A4 gene polymorphisms, reduce blood fentanyl concentrations in patients with burns. In this case, massive fluid loading was infused during the initial 24 h, and fluid loading continued for 24–48 h until the target urine output volume was achieved. These therapeutic interventions induce plasma protein dilution and intravascular volume expansion, thereby increasing the patient’s total fentanyl Vd. Han et al. and Kaneda et al. reported that when fentanyl was administered intravenously, the Vd of patients with burns was approximately twice as that of patients [5, 10]. However, these previous reports of fentanyl PK were demonstrated with a single bolus, and it was unclear for how long this Vd increase continued in patients with burns. Based on our observations, we found that the decrease in blood fentanyl concentrations continued until day 2, and the increase in Vd due to fluid loading might have remained 24–48 h after the burn injury. In fact, after day 3, when fluid resuscitation and sufficient urine volume were achieved, blood fentanyl concentrations tended to increase to near the predicted value.

Moreover, decreased blood fentanyl concentrations may also be affected by hypermetabolism associated with increased hepatic blood flow. Normal hepatic blood flow is 20 mL/kg/min, but it increases to 35 mL/kg/min in patients with burns in a hyperdynamic state [20]. Given that hepatic blood flow accounts for most fentanyl clearance, changes in hepatic blood flow after burn injury may affect the excretion kinetics [21, 22]. However, in patients with burns, the initial 36 h after the burn shows a hypodynamic stage associated with rapid fluid loss from the intravascular space and decreased cardiac output [23]. Subsequently, cardiac output increases, and the hyperdynamic state continues until the complete healing of the burn wounds [24]. Based on previous reports and our observations, the low blood fentanyl concentrations until day 6 may be related to the hyperdynamic state. However, the significant decrease in blood fentanyl concentrations during the burn shock stage may be due to increased Vd rather than the hyperdynamic state. Moreover, additional factors may explain enhanced fentanyl clearance in patients with burns. For example, debridement was performed on days 1 and 2. Bleeding due to surgical intervention may also affect the pharmacokinetics of fentanyl. However, the patient received chemical debridement by bromelain-based enzymatic debridement (Nexobrid) to minimize bleeding. Thus, the effect of bleeding associated with debridement on fentanyl PK was minimal. Moreover, some drugs, such as rifampicin and antiepileptics, induce CYP3A4 but the patient had no history of taking these drugs. Therefore, the decrease in blood concentration during the burn shock stage was considered to be related to the increased Vd associated with pathophysiological changes and therapeutic interventions.

Meanwhile, we were unable to measure the seeping amount of fentanyl through the burn wound. There was almost no measurable exudate until the blood concentration measurement point on day 2. Therefore, at this point, the effects of fentanyl leaking from the wound might be limited. Moreoever, we could not evaluate the hepatic blood flow in this patient. In our report, these factors have not been ruled out for the decreased blood fentanyl concentrations. In addition, fentanyl pharmacodynamics depend on the effect-site concentration (ESC) rather than the blood concentration, and it was necessary to clarify the ESC; however, there were no samples left for measurement. Based on the patient’s clinical course alone, we were unable to reveal a direct relationship between the decrease in fentanyl concentration and the increased Vd, and no other potential explanation could be identified. Further investigation of this phenomenon requires additional case reports and clinical data from the acute phase of burn injuries.

In conclusion, major changes in the fluid volumes of body compartments that occur with a large burn may increase the Vd of fentanyl, thereby lowering its concentration when a standard dose is administered. Our observations indicate that the PK of fentanyl in patients with severe burns can be substantially affected, especially during the burn shock stage, and suggest that it is important to adjust fentanyl administration rate depending on the patient’s findings, such as pain assessment and respiratory status, in this stage.

### Electronic supplementary material

Below is the link to the electronic supplementary material.

Supplementary Material 1

## Data Availability

Not applicable.

## Abbreviations

CYP: cytochrome P450
ESC: effect-site concentration
ICU: intensive care unit
NRS: numerical rating scale
PK: pharmacokinetics
