# Supplementary material for: Case report of pharmacokinetic analysis of continuous intravenous infusion of fentanyl in a patient with severe burn: burn shock stage complicates pain management
Source: J Pharm Health Care Sci. 2024 Jul 16;10:41. doi: 10.1186/s40780-024-00363-9 (PMC11251383; doi:10.1186/s40780-024-00363-9)
Supplement: Supplementary file 1 — Supplementary Material 1 [file 40780_2024_363_MOESM1_ESM.docx]

**Supplementary Figure 1**


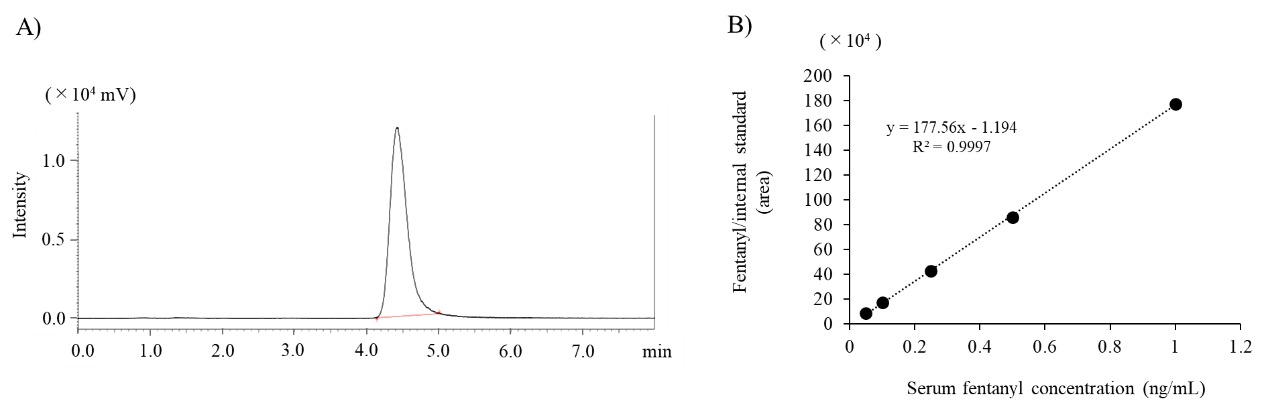


**Supplementary Figure 1.** The chromatogram of fentanyl in serum

The figures indicate that the chromatogram of fentanyl detected in our methods (A) and standard curve (B).
